# Possible Male Infanticide in Wild Orangutans and a Re-evaluation of Infanticide Risk

**DOI:** 10.1038/s41598-019-42856-w

**Published:** 2019-05-24

**Authors:** Cheryl D. Knott, Amy M. Scott, Caitlin A. O’Connell, Katherine S. Scott, Timothy G. Laman, Riyandi, Tri Wahyu Susanto

**Affiliations:** 1 Department of Anthropology, Boston University, 232 Bay State Road, Boston, MA 02215 USA; 2 Department of Biology, Boston University, 5 Cummington Mall, Boston, MA 02215 USA; 3 Department of Biological Sciences, Human and Evolutionary Biology, 3616 Trousdale Parkway, University of Southern California, Los Angeles, CA 90089 USA; 4 Department of Social Sciences, Oxford Brookes University, Oxford, OX3 0BP UK; 5 Department of Ornithology, Harvard Museum of Comparative Zoology, Harvard University, 26 Oxford Street, Cambridge, MA 02138 USA; 6 Department of Biology, Jl. Dr. Hadari Nawawi, University of Tanjungpura, Pontianak, 7812 Indonesia; 7 Department of Biology, National University, Indonesia, Jakarta, 1250 Indonesia

**Keywords:** Zoology, Animal behaviour

## Abstract

Infanticide as a male reproductive tactic is widespread across mammals, and is particularly prevalent in catarrhine primates. While it has never been observed in wild orangutans, infanticide by non-sire males has been predicted to occur due to their extremely long inter-birth intervals, semi-solitary social structure, and the presence of female counter-tactics to infanticide. Here, we report on the disappearance of a healthy four-month-old infant, along with a serious foot injury suffered by the primiparous mother. No other cases of infant mortality have been observed at this site in 30 years of study. Using photographic measurements of the injury, and information on the behavior and bite size of potential predators, we evaluate the possible causes of this injury. The context, including the behavior of the female and the presence of a new male at the time of the injury, lead us to conclude that the most likely cause of the infant loss and maternal injury was male infanticide. We suggest that in orangutans, and other species where nulliparous females are not preferred mates, these females may be less successful at using paternity confusion as an infanticide avoidance tactic, thus increasing the likelihood of infanticide of their first-born infants.

## Introduction

Infanticide, the intentional killing of an infant, is predicted to occur as a male reproductive tactic in situations where a male has a high probability that he has not sired a given offspring, a reasonable expectation that he may be able to successfully father the female’s subsequent offspring, and where premature loss of a suckling infant allows a female to conceive again sooner than she would have otherwise^1,2^. The definition of infancy varies between species but generally refers to the period in which an offspring is dependent on lactation to meet the majority of their caloric and nutritional needs. Infanticide has been reported in several mammalian orders, these include primates, carnivores, rodents, artiodactyles, equids and possibly cetaceans^3–5^. Amongst the primates, observed or suspected infanticide, and other attacks on infants, have been reported in 64 species, from both wild and captive populations^6–9^. However, even in those species where it is known to occur, individual instances are rare and the probability of an event being witnessed by a human fieldworker is thus slim^10^. Because infanticide happens quickly and is often performed by new males, who are thus more likely to be unhabituated^11^, it is often difficult to observe or is only inferred^12^.

Orangutan (*Pongo spp*.) infants are predicted to be vulnerable to infanticide due to a suite of life history and behavioral characteristics^3,13^. Two of these factors are non-annual breeding^4^ and a long period of lactation relative to gestation^3^. With a mean 7.6 year inter-birth interval, orangutans are non-annual breeders who have the longest period of lactation of any mammal^14–18^. Long periods of lactation result in extended lactational subfecundity, during which a female cannot conceive^19^. Thus, infant death will stop this period of lactational subfecundity, allowing a female to conceive again much more quickly than she would have otherwise^2,20^. As in humans^21,22^, maternal energetic condition, which is further depleted by the demands of lactation, likely also contributes to the long periods of lactational subfecundity in orangutans^19^. Orangutan females may also experience periods of high energetic stress due to fluctuations in food availability^19^. For these two reasons, orangutan females typically cannot conceive throughout the majority of their long inter-birth interval^19^. Thus, infanticide could be advantageous to orangutan males who have a low probability of paternity, since infanticide will stop the suppressive effect of suckling on female ovarian function as well as remove the energetic demands of lactation, thus shortening the period of lactational subfecundity. Amongst primates, infanticide is also most common in females that carry (as opposed to cache) their young and that do not receive significant forms of allomaternal care^3,19^. Orangutans also fit this pattern.

In addition to infanticide being attributed to male-male competition for paternity^23^, it is also considered a form of sexual coercion^24^. Sexual coercion is the use of force, or threat of force, by a male to increase the chance that a female will mate with the coercive male when she is fecund, or decrease the chance that she will mate with another male, at some cost to the female^24^. While infanticide does not necessarily cause physical harm to the female herself, it is costly for a female, as she loses significant reproductive investment with the death of her infant^24^. One of the factors selecting for male coercion of females is a female’s lack of allies^24^. Orangutans are semi-solitary, with females spending long periods of time alone, thus females are rarely in the presence of other adults, specifically potential sires of the infant that could potentially intervene, or come to her aid, during an infanticidal attack. These two factors, long periods when a female cannot conceive and a semi-solitary lifestyle, are also significant contributors to forced copulation in orangutans, another form of sexual coercion that is particularly prevalent in this species^24–26^, with rates variable depending on the study site^25^. Despite possessing these risk factors, infanticide has not yet been observed in wild orangutans, and so far, no suspected cases have been reported. In fact, infant loss is extremely rare in orangutans^17,27^. The only confirmed incidence of orangutan infanticide comes from captivity where an adult male killed a newborn infant, albeit one that he had sired, apparently an aberrant behavior due to captivity^28^.

In many anthropoid primates, females have evolved a variety of counter-tactics to decrease the likelihood of male infanticide^24^. These counter-tactics involve physiological responses or adaptations, maternal aggression, avoidance of infanticidal individuals, social alliances, and strategic or multi-male mating^2,29,30^. These tactics largely apply to protection against infanticide once the threat is present, but female mating tactics can reduce the risk of infanticide before the infant is born. Promiscuous mating leads to paternity confusion, so that many males could be the possible sire of an infant^2,31^. It may be in a female’s best interest to mate promiscuously, and sometimes conspicuously, to give males an inflated estimate of paternity certainty^32^. In species with concealed ovulation, females can mate during non-fertile periods and post-conception to obfuscate paternity even further^2,33^.

Female orangutans exhibit some of these physiological and behavioral adaptations that are consistent with infanticide avoidance. Physiologically, orangutan females do not exhibit visual displays of receptivity, such as sexual swellings, but they display a white or reddish labial swelling near the end of the first trimester of pregnancy that could serve to inform males that copulation will not result in conception^34–37^. Female orangutans normally prefer to mate with prime, flanged males. Genetic paternity analysis from three orangutan sites in both Borneo and Sumatra also shows that flanged males sire more offspring than unflanged male (23 offspring sired by 9 flanged males compared to 11 offspring sired by 6 unflanged males)^38–40^. However, unflanged males achieve more matings than flanged males^25,34,41^. This is, at least in part, due to females preferentially mating with unflanged males when conception risk is low, a behavior consistent with the anti-infanticide tactic of paternity confusion^35^. Additionally, pregnant female orangutans are highly proceptive to non-prime males^35^. Such post-conceptive mating is also interpreted as a way to confuse paternity and is common in species that are at risk of infanticide^42^.

Female parity is also expected to influence infanticide risk, as two female counter-tactics may improve with greater maternal experience: maternal protective behavior and paternity confusion. First, experienced mothers may be better at avoiding potentially infanticidal situations. Thus, if maternal protection influences the occurrence of infanticide, then infants of primiparous females will be more vulnerable to infanticide than infants of multiparous females^43^. However, there is mixed evidence for and against the hypothesis that primiparous mothers are more vulnerable to infanticide. In blue monkeys, parity was not found to be a significant factor in determining the incidence of infanticide during a male takeover^43^. In contrast, primiparous chacma baboons were more likely to lose infants to infanticide than multiparous females^44^. In gorillas, Fossey^45^ found that primiparous gorilla mothers are three times more likely to lose infants to infanticide than multiparous mothers because primiparous females are more likely to transfer groups. Watts^45^ also found that more first born gorilla infants were victims of infanticide, however, the difference was not statistically significant. In these gorilla and blue monkey studies, female promiscuous mating may not be a viable counter-tactic due to the primarily one-male social systems of these populations.

Here we report on the case of a primiparous female orangutan (*Pongo pygmaeus wurmbii*) who received a serious injury, losing part of her left foot, coincident with the disappearance of her 4-month-old infant. This reported case is the only known or suspected occurrence of infant mortality in over 30 years of orangutan research at Gunung Palung National Park. We describe the injury and review the possible causes, arguing that sexually selected male infanticide is the most likely explanation for both her infant loss and the injury she sustained. We argue that her mating and social behavior, before and after the infant loss, are consistent with suspected infanticide by a male. We also present data on the female’s general condition after the injury, including results from urinalysis, controlling for food availability. It has been argued that infanticide is not a sexually selected tactic in male orangutans because it is unlikely that an infanticidal male will still be in the vicinity of a female long enough after killing her infant for the female to return to cycling^46^. However, we provide observations to the contrary in this paper. Finally, we suggest that aside from maternal experience^47^, primiparous females may have increased vulnerability to infanticidal attacks on their infants because they are generally not as attractive as parous females and thus are hampered from using paternity confusion as an anti-infanticide tactic.

## Methods

### Study site and behavioral observations

This study was conducted at the Cabang Panti Research Station in Gunung Palung National Park, located in West Kalimantan, Indonesia, on the island of Borneo. The current project was established in 1992, with long-term data collection beginning in 1994, and a data gap between 2003 and 2008. Between August 1994 and December 2015 over 71,000 hours of observation were conducted on orangutans in this study population. Orangutans were followed through focal animal sampling from night-nest to night-nest, or until they were lost. It was not possible to record data blind because our study involved known focal animals in the field.

The subject of this study is the adult female orangutan, Walimah, who was born on October 30, 1998. At the time of the injury reported here Walimah was 16.6 years old. This female is extremely habituated to human presence. Since her birth, through December 2015, she was followed for 540 days (5620 hours) of focal observation, 582 days (6245 hours) where she was observed when her mother was the focal individual, and 355 days (3834 hours) when she was in association with another orangutan as a non-focal individual. Thus, she had spent at least 1477 days and 15,699 hours in the presence of humans at the time of this study. Her follows were distributed across all years and periods of food availability.

Standard orangutan data collection protocols^48^ were used alongside established methods of recording nutritional intake, energy expenditure, and behavior^49^. The position of the infant on the mother’s body was recorded every 5 minutes as well as any distance separation between mother and infant. During orangutan follows, every orangutan within 50 m of the focal animal was recorded and considered to be in association. Social interactions and mating events were recorded on an *ad libitum* basis and an additional data check-sheet was filled out when mating was observed. We defined proceptive behaviors as sexual behaviors performed by the female that invite, facilitate, or achieve sexual contact with the male. The same data collection protocols have been used since the beginning of the study and thus we compared Walimah’s mating behavior to our database records from other females at Gunung Palung, collected between January 1997 and December 2015. We limited our comparisons of mating behavior to females who had greater than 180 hours of focal animal observation during the peri-conceptive period. We define the peri-conceptive period as one year prior to conception through the first trimester of pregnancy. This is when females show the greatest interest in mating and encompasses the mean waiting time of 4–10 months between the resumption of mating after giving birth to the next conception^15,19^. Orangutans have concealed ovulation, and pregnancy status is effectively not revealed to males until approximately the end of the first trimester when they display a white or reddish labial swelling^34^–^37^.

### Fruit availability

Fruit availability was determined from monthly monitoring of orangutan fruit trees (>14.5 cm diameter at breast height) and lianas (>3.5 cm diameter at breast height) in 70 plots (20 m × 50 m) and belts (100 m × 20 m) established and monitored by A. Marshall^50^, and his assistants, in collaboration with CDK. From this dataset the kilocalories of orangutan fruit available per hectare^51^ were estimated for each month based on the top 25 orangutan fruit genera established from 23, 232 feeding observations collected between 1994 and 2015. This measurement is calculated by summing, for each genus, the # stems/hectare × percentage of stems fruiting × mean fruit crop size/stem × mean grams/fruit × mean kcal/gram = Kcal available/hectare^51^. For statistical comparisons, Kcal available/hectare were converted to a Z score for each month, based on monthly estimates of food availability from 1994–2016, standardizing across study periods as described above and in Knott^49^.

### Urine collection and analysis

Urine samples were collected whenever possible, particularly from first-morning voids during focal follows^51^. Samples were collected either using a plastic sheet or were pipetted directly from vegetation^52^. Samples contaminated by feces were discarded. Chemical reagent strips (Boehringer Mannheim Chemstrip 10 with SG) that test for the presence of disease and physiological status were used to assess the presence of ketones in urine as an indicator of weight loss from the breakdown of fat stores and leukocytes as an indicator of infection^49,52^. Pregnancy was tested using the OneMedhCG Urine Pregnancy Test that measures HCG (Human Chorionic Gonadotropin).

### Museum measurements

We used a mounted museum specimen of an adult Bornean female orangutan at Harvard University’s Museum of Comparative Zoology (MCZ Catalog #5974) to aid in estimating the approximate size of the bite wound observed. We took multiple photographs of the feet of the mounted specimen with a tape measure placed in the photos for comparison. These photographs were sized and rotated in the computer to match photographs of the injury (taken on July 30, 2015). This allowed us to obtain an estimate of the minimum size of the wound. We also measured the maxillary and mandibular inter-canine distance of available male and female orangutan specimens from the MCZ as well as two adult male orangutan specimens from Gunung Palung. Inter-canine distance was measured in two ways, as the distance between the cusp tips of the canines (ICD1) and as the greatest distance between the distal surfaces of the canines (ICD2).

### Infanticide literature review

We reviewed the published accounts of infanticide in apes, and recorded how often infanticides were observed or inferred in order to put our observations in context. Mothers are sometimes injured during infanticidal attacks^53^. Thus, replicating the protocol of van Schaik^53^ we reviewed infanticide cases in the primate literature to determine the incidence and location of female injuries in order to better understand the probability of female injury during an infanticide. If a detailed description of the attack was given, but no injuries were mentioned, we assumed that no injury occurred. If the infanticide report did not contain a detailed description of the attack, it was labeled as insufficient data.

### Statistical analyses

To assess the impact of Walimah’s injury on physiological markers in her urine, we used a GLMM with leukocyte level as the target variable, injury status (before or after) as a fixed effect and relative food availability as a random factor. Differences in sociality across periods were assessed using GLMM. The reproductive/injury status was treated as a fixed effect and observer as a random factor.

### Ethical approval

All applicable international, national, and/or institutional guidelines for the care and use of animals were followed. All procedures performed in studies involving animals were in accordance with the ethical standards of the institution or practice at which the studies were conducted. This research was approved by the Standing Committee on the Use of Animals in Research and Teaching at Harvard University, Protocol no. 95–04 and by Boston University’s Institutional Animal Care and Use Committee (IACUC), Protocol numbers 11-045 and 14-043.

## Results

### Reproductive behavior and birth

The first time Walimah was observed mating was a forced copulation with an unflanged male on April 27, 2009, when she was 10.4 years old. Despite many hours of observation (1846 hours), she was not seen mating again until March 20, 2014 when she was 15.4 years old. During this interim period, she spent 60% of her social time at a median distance of 17.3 m from adult flanged or unflanged males, but without mating (51% of her social time was exclusively with males – without any females present). Starting in August 2013, however, Walimah began following and engaging in proceptive behavior with the flanged male Codet. During these interactions, Codet would remain motionless while Walimah closely inspected him, including his penis, lightly touched him and presented her genitals to him. After 7 months of such interactions, with no mating observed, Walimah successfully mated with Codet after 26 minutes of prolonged manual and oral stimulation and attempted intromission by Walimah. These behaviors re-occurred over the next two days. Such mating interactions, involving extensive proceptive behavior by the female, but with no male mating attempts, and intense focus on one flanged adult male, have also been reported in other studies of primiparous, adolescent females^34,54,55^ but are not observed in multiparous females. Post-conception, Walimah traveled with an unflanged male, with whom she mated twice, once cooperatively and once forced. These matings did not involve the elaborate and prolonged ‘foreplay’ by Walimah that was seen with the flanged male Codet.

Table 1 shows that during Walimah’s peri-conceptive period, she was observed traveling with 8 individually recognizable flanged or unflanged males and an additional 1 to 4 unflanged adult males who were not individually recognizable. During this same period of time, there were 18 individually recognizable adult males seen in the study site and an additional 3–9 adult males who were not individually recognizable. Walimah was only observed to mate with two of these males. Despite spending time with at least 9 different adult males, she concentrated her mating effort on the flanged male Codet.

**Table 1:** Walimah’s mating effort during her peri-conceptive period (7/9/2013–9/25/2014).

Male	Status	No. Days Encountered	Time in Association (min)	No. Matings	Female Ovulatory Status	Duration of Sexual Contact (min)	No. Sexual Behaviors	No. Female Proceptive Behaviors	Nature of Mating
Codet	Flanged	14	4199	4	Peri-ovulatory	84	104	67	highly proceptive
Malik	Unflanged	9	4045	2	Post-conception	28	39	21	1 forced, 1 cooperative
Yoda	Unflanged	3	941	0	NA	0	0	0	NA
Kecil	Unflanged	3	934	0	NA	2	2	0	NA
Balu	Flanged	3	657	0	NA	0	0	0	NA
Syklops	Unflanged	3	131	0	NA	0	0	0	NA
Zorro	Unflanged	1	455	0	NA	0	0	0	NA
Prabu	Flanged	1	141	0	NA	0	0	0	NA
Unknowns	Unflanged	4	332	0	NA	0	0	0	NA

Walimah’s first pregnancy was confirmed on July 18, 2014 based on detecting HCG in her urine via a urinary test strip. Mean length of orangutan gestation has been measured as 245 days^56^. Based on the finding that HCG is detected, on average, 9 days after conception in humans^57^ and that pregnancy hormone production in orangutans is remarkably similar to humans^58^, we estimated her conception date as July 9, 2014 and her due date as March 1, 2015.

**Figure 1:**
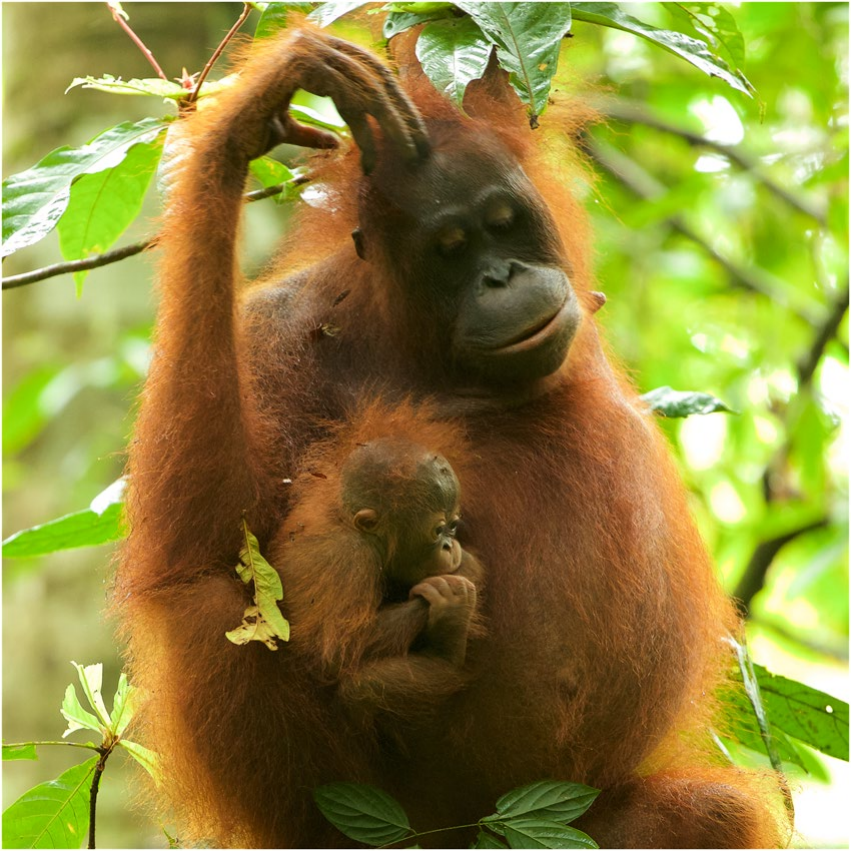
Walimah with infant, approximately 37 days old, taken on April 7, 2015. Photo © Tim Laman.

Walimah was found with her newborn infant on March 11, 2015. The infant was judged to be 1–2 weeks old, giving her an estimated birth date of approximately March 1, 2015, as predicted. Infant sex was determined to be female through photographs and visual observation and confirmed independently by multiple observers. Walimah was followed with her new infant for 35 days until they were lost on April 15, 2015. During this time period she showed species-typical maternal behavior. The infant was carried, via clinging and sometimes maternal support, for 100% of observation hours during this period. The infant appeared strong, alert, and healthy the entire time (Fig. 1).

### Infant loss and foot injury

On July 6, 2015, Walimah was discovered again after a period of 82 days with no observation. Her infant was no longer present. Walimah’s home range was searched for any sign of her infant, but no remains were found. Given the infant’s total dependence on the mother at this stage, the infant was presumed dead. Based on the birth date and the time of discovery, the age of death of the infant was estimated as between 1.5 and 4 months old. This represents the first known infant orangutan death since the establishment of the study site in 1984.

**Figure 2:**
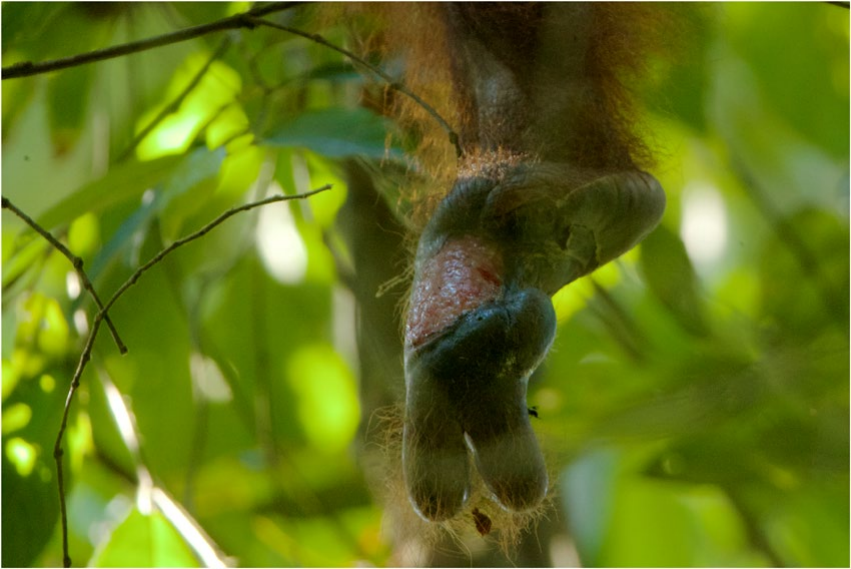
Injury to left foot, taken on July 30, 2015. Photo © Tim Laman.

Concurrent with the infant loss, Walimah was seen on this date to have a severe injury to her left foot (Fig. 2). The wound was estimated to have occurred 1–2 weeks prior, based on the state of the exposed tissue and the subsequent monitoring of wound healing. Walimah’s wound showed that a large portion of her foot was missing, as well as the fourth and fifth toes. Her remaining second and third digits were swollen and bent under her foot and she was no longer capable of using these digits. She retained her heel, however, and first toe. Additionally, her hair and possibly some tissue on the top layer of her foot were also missing. This area appeared red and raw and she habitually licked the wound. No other injuries were observed.

Walimah also appeared to be visibly thinner than when she was last observed. However, Chemstrip analysis of her urine (n = 91) did not reveal the presence of any ketones after her injury. Thus, we can conclude she was not experiencing significant weight loss. However, the mean leukocyte level (n = 91) during her post-injury period was significantly higher than at any time during the two years prior to her injury (F = 5.147, df = 1, p < 0.05).

Over the course of the following month, the swelling on digits 2 and 3 decreased, yet they remained bent and were not used. We note that this was the first injury to a female orangutan that we witnessed at Cabang Panti^59^. This is in contrast to multiple and extensive injuries observed in males including missing eyes, damaged flanges, injured fingers and toes, and deep back wounds. In our analysis of wounding at Cabang Panti, of 17 known injuries, except for this case, all victims were male, 71% flanged and 23% unflanged^59^.

### Subsequent mating and social behavior

Twenty-three days after Walimah was found injured, and with her infant missing, she was seen in association with an unflanged male who was new to the study population. This male was first observed in the study site two weeks before Walimah gave birth and she had no prior social interaction with him. Her first recorded mating after giving birth was with this same unflanged male on November 8, 2015, four months after she was found with her injury. It is possible that she mated earlier than that as she was seen in association with a male during 40% of her follow days during that period and was not followed continuously. In November 2015, she was observed mating with two different males.

**Figure 3:**
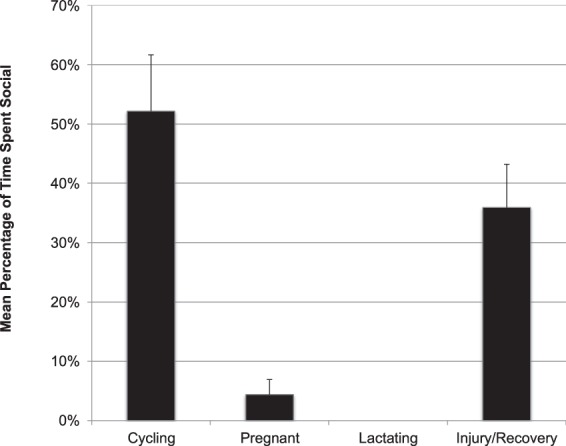
Comparison of percentage of her awake time, during full day follows, that Walimah spent in association with other orangutans when she was cycling (n = 22), pregnant (n = 47), lactating (n = 35) and after she was injured/recovering (n = 36).

In order to assess whether Walimah’s social behavior changed dependent on her reproductive or injury status, we examined data collected between August 1, 2013, when she first started consorting with adult males, to February 28, 2016. This incorporated the recovery period after her injury and infant loss. We restricted our analysis to full day follows in order to be able to compare the daily rate of sociality. This gave us a sample of 140 full days, comprised of 15.7% of days cycling, 33.6% of days pregnant, 25.0% of days lactating and 25.7% of days during her injury/recovery phase. Figure 3 compares the time she spent in association with other orangutans during these four periods. The percentage of time spent social was significantly influenced by reproductive/injury status (F = 14.4, df = 3, p < 0.001). Overall, 90% of her time spent social was in association with a male. Walimah had no social interactions when she had her lactating infant. Results of the GLMM, showing estimated mean differences between periods, indicate that the percentage of time spent social during her post-injury phase was not significantly different than her earlier cycling phase (estimate = −0.13, p = 0.131), but she spent significantly more time social during her injury phase compared to her lactation (estimate = 0.32, p < 0.001) and pregnancy (estimate = 0.28, p < 0.001) phases. During lactation, she spent significantly less time social than when she was cycling (estimate = −0.46, p < 0.001), but lactation and pregnancy levels of sociality were not significantly different (estimate = −0.05, p = 0.46). She also spent significantly less time social during pregnancy compared to during cycling (estimate = −0.41, p < 0.001).

### Comparison with museum specimens

**Figure 4:**
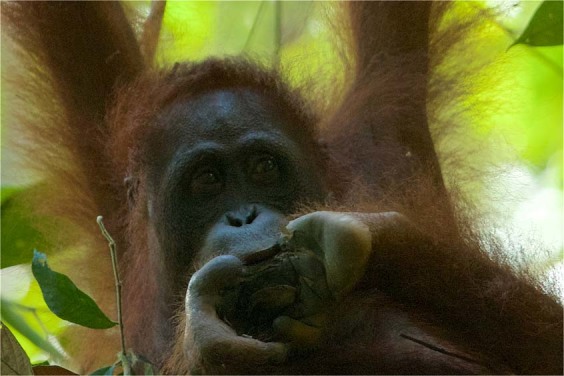
Comparison of foot injury to orangutan mouth size. Photo © Tim Laman.

**Figure 5:**
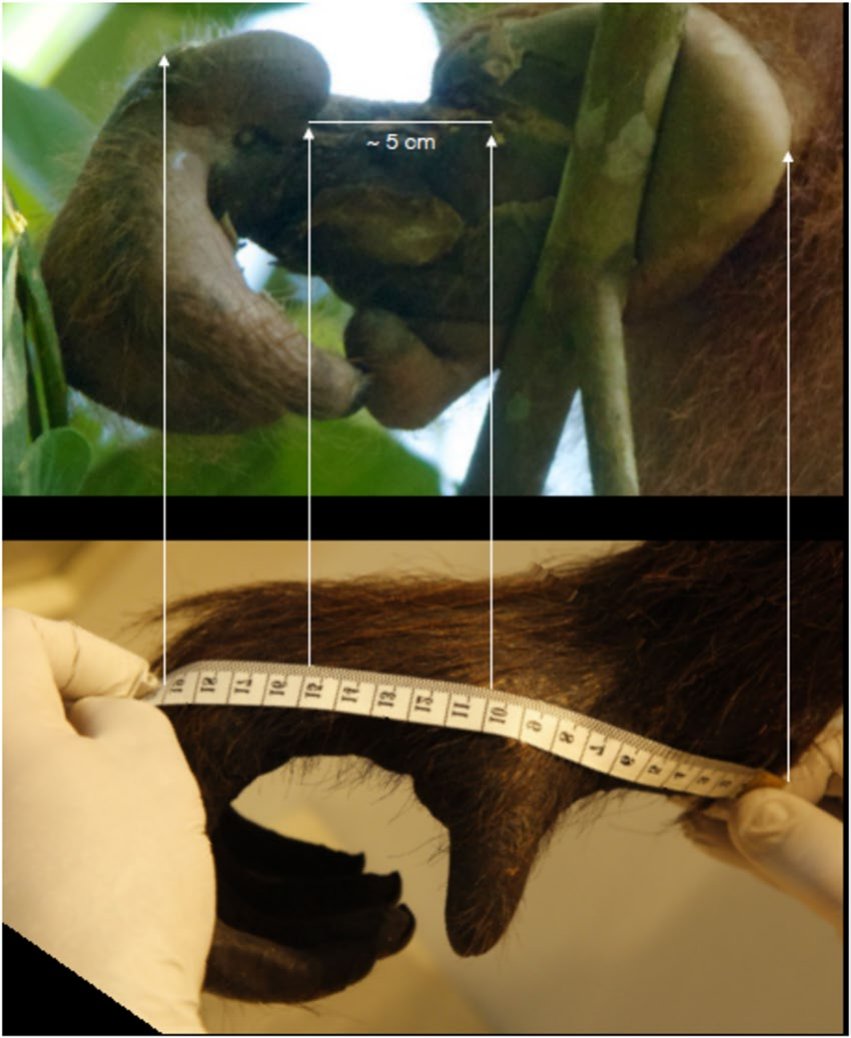
Comparison of Walimah’s foot injury to museum specimen. Photo © Tim Laman.

We used three different close-up photographs of the foot of the mounted female orangutan specimen, positioned next to the measuring tape, and compared them to two different photographs of Walimah’s injured foot, taken at different angles. The shape of the wound is consistent with a severe bite removing a portion of her foot. Figure 4 shows Walimah licking her wound and the size of the wound in comparison to her own mouth. Field observations and video revealed that her jaw fit into the gap in her foot as she was observed to lick the inner part of the wound that was still unhealed. The wound was swollen, so the fact that her jaw fit into the wound indicated to us that the injury was caused by a jaw at least as big, or likely bigger, than Walimah’s. Given the body position of the mounted orangutan specimen, it was not possible to photograph the foot from below, as with the wild orangutan. However, by rotating the photographs and sizing them to match, the width of her wound was estimated as 5 cm (Fig. 5). The same wound width result was obtained on all 3 photographs. As her foot was swollen, 5 cm was considered the minimum wound width. Our measurement of the inter-canine distance of the maxilla and mandible of male and female orangutan specimens are shown in Table 2, with male maxillary ICD ranging from 6.27–8.32 cm and female maxillary ICD ranging from 5.32–6.33. Thus, the estimated bite wound size of larger than 5 cm falls within the orangutan maxillary ICD range.

**Table 2:** Mean inter-canine distances (ICD) of Bornean orangutans.

	ICD1 (cm)			ICD2 (cm)		
	mean ± sd	range	n^a^	mean ± sd	range	n^a^
Male maxilla	6.27 ± NA	NA	1	7.49 ± 0.61	6.77–8.32	5
Male mandible	5.49 ± 0.35	5.24–5.74	2	5.95 ± 0.51	5.39–6.72	6
Female maxilla	5.55 ± 0.37	5.32–5.98	3	5.89 ± 0.33	5.62–6.33	2
Female mandible	4.25 ± 0.24	4.07–4.41	2	4.58 ± 0.30	4.16–4.85	4

^a^Sample size varies due to the condition of the canines in the specimens measured.

### Literature review

**Figure 6:**
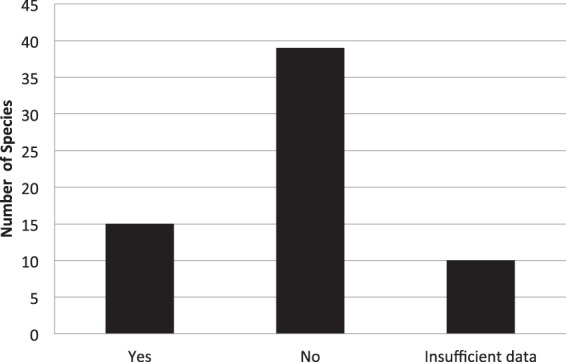
Incidence of maternal injury across primate species during an infanticidal attack.

We reviewed 113 papers on primate infanticide to determine prevalence of maternal injury during an infanticidal attack (Fig. 6). Of the 54 species that had sufficient data available to determine if mothers were injured, there was a report of at least one mother injured in 15 species. Table 3 shows the location of each case of maternal injury concurrent with an infanticidal attack by a male. It is worth noting that mothers are injured during infanticidal attacks in other great ape species. In the majority of the cases in which the location of the maternal injury was given, the injury occurred on the female’s extremities (8 out of 12). Additionally, our review of the cases of infanticide by male apes revealed that 51% of all such cases were observed and 49% were inferred based on the co-occurrence of the disappearance of a healthy infant and a social encounter with a potentially infanticidal male, such as a new immigrant or an intergroup encounter (Table 4). However, it should be noted that for all of these species, except *Hylobates lar*, at least one infanticide by males has been observed.

**Table 3:** Incidents of maternal injury (each line represents a single mother) in primates concurrent with an infanticidal attack by a male.

Species	Location of Injury	Reference
*Tarsius spectrum*	bite, no location given	Gursky-Doyen^7^
*Alouatta caraya*	hair missing from back	Pave *et al*.^133^
*Alouatta seniculus*	no location given	Crockett^12^
*Alouatta seniculus*	no location given	Crockett^12^
*Alouatta seniculus*	no location given	Crockett ^12^
*Cebus capucinus*	cheek	Manson *et al*.^134^
*Cercocebus atys*	large infected wound on leg	Fruteau *et al*.^135^
*Cercopithecus campbelli*	no location given	Galat-Luong and Galat^136^
*C. mitis stuhlmanni*	no location given	Cords and Fuller^43^
*Macaca radiata*	right hand	Singh *et al*.^137^
*Papio anubis*	no location given	Collins *et al*.^138^
*Papio cynocephalus*	face, tail, forelimbs, hand	Shopland^139^
*Papio hamadryas*	neck bites	Swedell and Tesfaye^140^
*Papio ursinus*	left arm	Palombit *et al*.^44^
*Theropithecus gelada*	limb	Mori *et al*.^141^
*Theropithecus gelada*	palm	Mori *et al*.^141^
*Gorilla gorilla beringei*	head wounds, possibly fatal	Fossey^142^
*Pan troglodytes*	eye, cheek, hand	Murray *et al*.^130^
*Pan troglodytes*	face, ears, fingers, toes	Wilson *et al*.^143^

**Table 4:** Observed and inferred cases of infanticide by males in apes.

Species	Reference	Observed	Inferred	Total
*Hoolock hoolock*	Alfred and Sati^144^	1	0	1
*Hylobates lar*	Borries *et al*.^145^	0	1	1
*Gorilla beringei beringei*	Fossey^142^	3	6	9
*Gorilla beringei beringei*	Watts^47^	0	8	8
*Gorilla beringei graueri*	Stokes *et al*.^146^	0	3	3
*Gorilla gorilla gorilla*	Yamagiwa and Kahekwa^147^	3	0	3
*Pan troglodytes*	Arcadi and Wrangham^131^	1	0	1
*Pan troglodytes*	Bakuneeta *et al*.^148^	0	1	1
*Pan troglodytes*	Boesch *et al*.^149^	1	0	1
*Pan troglodytes*	Bygott^150^	1	0	1
*Pan troglodytes*	Goodall^106^	2	1	3
*Pan troglodytes*	Hamai *et al*.^151^	2	0	2
*Pan troglodytes*	Kawanaka^152^	0	1	1
*Pan troglodytes*	Masui in Hamai *et al*.^151^	1	0	1
*Pan troglodytes*	Murray^130^	1	0	1
*Pan troglodytes*	Newton-Fisher^153^	1	1	2
*Pan troglodytes*	Nishida *et al*.^154^	0	2	2
*Pan troglodytes*	Nishida and Kawanaka^155^	0	1	1
*Pan troglodytes*	Norikoshi^156^	0	1	1
*Pan troglodytes*	Sherrow and Amsler^157^	3	0	3
*Pan troglodytes*	Suzuki^158^	1	0	1
*Pan troglodytes*	Takahata^159^	1	0	1
*Pan troglodytes*	Watts and Mitani^160^	2	0	2
*Pan troglodytes*	Watts *et al*.^161^	1	0	1
*Pan troglodytes*	Wilson *et al*.^143^	2	0	2
**Total**		**27**	**26**	**53**

### Nulliparous female mating behavior

We also considered whether Walimah may have been more vulnerable to infanticide because she was a primiparous mother and thus examined her mating behavior when she was nulliparous. Walimah was only observed mating with 2 different males or 0.38 unique male mating partners/100 observation hours during her peri-conceptive period. This is lower than the mean for parous females. Parous females (n = 5) were observed mating with an average of 2.6 males or 0.76 unique male mating partners/100 observation hours during their peri-conceptive periods. Nulliparous females (n = 2) mated with an average of 1.5 males or 0.32 unique male mating partners/100 observation hours during their peri-conceptive periods. Although our sample size was not large enough to test for statistical significance, these differences are potentially meaningful in terms of promiscuous mating and the female’s ability to confuse paternity.

## Discussion

### Possible causes of injury

Although the exact circumstances of Walimah’s injury and loss of her infant can never be positively determined, we can evaluate both the possible and the probable causes of these events. Given that the loss of an infant orangutan and a severe injury to a female are both extremely rare, they occurred very close in time, and neither has been seen in over 30 years of research at this site, we argue that it is most parsimonious, without any evidence to the contrary, that these events occurred at the same time, due to the same cause. However, we cannot rule out the possibility that this injury and infant loss were separate events.

### Mechanical injury

We ruled out the possibility that Walimah’s injury was caused by a mechanical injury such as being struck by a falling tree or branch or falling out of a tree. While such things are possible, but unlikely, events for orangutans, such an injury would lead to a fracture wound, not the loss of a large chunk from the middle of her foot. There are also no known cases of other kinds of mechanical injuries to orangutans caused by environmental hazards.

### Sunbear

Gunung Palung National Park hosts a population of sun bears (*Helarctos malayanus*). These are the smallest bears in the world, weighing 25–65 kg^60,61^. They are opportunistic omnivores, primarily eating fruit, and augmenting their diet with stingless honey bees, termites, ants, larva, honey and small mammals^62–64^. Sunbears travel on the ground and are able to forage arboreally^60^. Although the mean width of sun bear canines (6.08 cm)^65^ is large enough to deliver the wound we observed, a sun bear attack seems highly unlikely for the following reasons. First, there are no known attacks of sunbears on orangutans^61,62,66–68^. In fact, we were unable to find any accounts of sunbear attacks on any large animals except for humans, either on the ground or in the trees^61,62,66–68^. Sun bears are normally passive, but there have been occasional attacks on humans when surprised^69–71^. Second, this kind of wound is not consistent with a sun bear attack. They have poor eyesight and attacks on humans typically happen when they are startled at close range^69–71^. One such attack on a human occurred at our research site in 2000 and involved a canine puncture wound. Walimah showed no puncture injuries. Furthermore, attacks typically also involve scratches from the sun bears’ sharp claws, and scratches were not present on Walimah. Third, an orangutan that did encounter a sun bear could easily escape vertically, being a far superior climber. Although they are arboreal, sun bears claw their way up and down tree trunks vertically^60,72^. They are unable to swing or jump between trees^73^. Orangutans, however, are extremely adept arboreal climbers and are easily able to move between trees. Thus, an orangutan confronted by a sun bear in the canopy could easily escape. Therefore, we conclude that the nature of the injury, the lack of any prior attacks on orangutans, and the typical behavioral patterns observed in both sun bears and orangutans do not support a sun bear as a likely cause of this injury.

### Clouded leopard

The largest feline predator in Borneo is the clouded leopard (*Neofelis diardi borneensis*)^74^. Clouded leopards are medium-sized felids, ranging from 11 to 23 kg in weight^75,76^, that typically prey on small animals, such as juvenile bearded pigs, small deer, pangolins, monkeys and squirrels. Clouded leopards, like most cats, kill by stalking their prey, and jumping on them from behind^73,77^. Prey are dispatched by delivering one killing bite to the neck or back, with their long canines severing the spinal cord^78–80^. Once a bite is made, the cat typically holds on until the animal dies^73,80^. This is opposed to canids that deliver multiple, slashing bites^79^. Clouded leopards have the longest canines for their body size of any cat and their canines have the highest breaking point^79–81^. Their small size and slender canines give them the weakest bite force (344.2 N) of any of the medium to large-sized felids^79–81^. Large cats have much greater bite forces (e.g. 1234.3 N in the tiger, *Panthera tigris*)^80,82^. Clouded leopard attacks involve puncture wounds on the spine, at the back of the neck, as well as claw scrapes^75,77^. The puncture wounds on one animal, known to have been killed by a clouded leopard, had an inter-canine width of 3 cm^75^. Clouded leopard attacks on infant and juvenile proboscis monkeys (*Nasalis larvatus*) consist of bites to the necks, delivered from behind^83^. Another reported clouded leopard attack involved a juvenile, 3.7 kg, siamang (*Symphalangus syndactylus*) with a severed vertebrae at C2^84^. There are no reported cases of clouded leopard attacks involving wounds other than on the neck or back^73,75–78,85,86^.

There are no observed cases of clouded leopards attacking orangutans, but there are several suspected cases. One juvenile orangutan died of injuries that were suspected of being caused by a clouded leopard^87^. This attack was not witnessed, but the authors argue that a clouded leopard is implicated due to two puncture wounds on the back^87^. At Tuanan Research Station, an adult female orangutan is suspected of having been killed by a clouded leopard^18^. However, this was an atypical female who lost her home range due to mining, logging, and fires and regularly traveled on the ground^88^. Her unusually high level of ground travel would have made her vulnerable to such an attack. The nature of the injuries that led to this suspicion have not been described. Additionally, Rijksen^89^ reports that 7 juvenile, ex-captive, rehabilitant orangutans on Sumatra, each weighing less than 10 kg, were apparently killed by a clouded leopard while walking on the ground around the feeding station. Given the nature of the attacks, Rijksen^89^ concluded that clouded leopards are too small to successfully prey on larger orangutans and that juveniles accompanied by their mothers would be protected from attack. He also attributes these deaths to one individual clouded leopard that had discovered the vulnerability of these rehabilitant juveniles at the feeding station. We could find no other reports of attempted or successful clouded leopard attacks on orangutans^73,76–78,85,86^.

Both the location and nature of Walimah’s injuries are thus inconsistent with a clouded leopard attack for the following reasons. First, clouded leopards stalk their prey and then attack from the back, delivering puncture wounds to the head, neck and upper extremities^90,91^. Previously known or suspected cases of clouded leopard attacks on orangutans involved such injuries. Walimah’s injury did not involve a puncture wound and was delivered to the foot. Second, the canine width of clouded leopards is significantly smaller than the estimated width of Walimah’s injury. The injury to Walimah’s foot was estimated to be a minimum of 5 cm wide, significantly larger than the 3 cm inter-canine width of a clouded leopard. Third, the clouded leopard is a small cat that kills successfully by using their extremely long canines to sever the spinal cord. They have a weak bite force and thus are unlikely to have the jaw strength to bite through and remove bone from an orangutan’s foot.

### Human

Another possible source of attack is from a human. Humans do attack and kill female orangutans to take their infants^92^. However, we do not suspect that this was a human attack. First, all known cases of humans taking infants involve the use of guns of some sort^93^, and the mother is killed. Because the infant was being carried on her body, the only way a human would have been able to take this infant would have been by shooting the mother and then taking the infant after the mother fell from the tree. There is no evidence that Walimah had any injuries associated with being shot or having fallen. Hunters, or local people, may carry a *parang* (machete) into the forest. But, Walimah’s injury does not involve a cut or slice that could have been delivered by a *parang*. We also note that there are no metal animal snares found in this forest, as are seen in some African forests^94,95^ and thus we can also rule out a snare injury.

### Orangutan

The only source of recorded injury for wild orangutans at this study site are adult male orangutans. Orangutan males are known to attack and wound other males^96,97^. In our review of orangutan wounding patterns at Gunung Palung^59^ we found that 81% of males, who had been followed for at least 5 days, had sustained orangutan-inflicted wounds. Of these, 35% were to the hands and feet. Male orangutans often are seen with bent or missing digits caused from bites delivered by other males^96,97^. Furthermore, caged male orangutans have been known to bite off the fingers of their human keepers. Thus, male orangutans regularly bite the hands and feet of other male orangutans and are capable of biting off pieces of flesh and bone. We suggest that this is what may have happened to Walimah as she fought with a male during an attack that was aimed at killing her infant.

The nature of the wound itself is also consistent with an orangutan attack. We estimated the wound as being at least 5 cm wide (Fig. 5), within the range of orangutan canine breadth (Table 2). An adult male orangutan would also have the strength and ability to deliver such a wound. Orangutan males, both flanged and unflanged, sometimes forcibly copulate with females^25,37,98^. Although forced matings are not known to result in serious injury to females^25^, they do indicate that both flanged and unflanged male orangutans are able to restrain and overpower females^46^. Finally, orangutans were calculated to have a bite force of 2560 N at the 2^nd^ molar (the position where bite force was measured)^99^, an order of magnitude greater than the 344N bite force of the clouded leopard at the canine or 547 N at the carnassial (the 4^th^ upper premolar and 1^st^ lower molar)^80,100^. It is worth noting that the bite force of an orangutan is far greater than even a tiger. Orangutans have powerful bites because some of their foods, particularly seeds, are extremely hard and they feed by crushing these food items^101^. Cats on the other hand, kill by puncturing with their canines, and feed by tearing, not crushing their prey.

Walimah’s injury represents the first known wounding attack on a female orangutan at Gunung Palung. Attacks and injuries to female orangutans, in general, are very rare^102^. However, such attacks have been reported from Tuanan Research Station^102^ where it has recently been reported that there was a fatal coalitionary attack by a young female and an unflanged male on an older resident female who had a four-year-old offspring^88^. The authors argue that although the young female initiated the attack, it proved lethal when she was joined by an unflanged male, who delivered the most severe injuries, including a serious bite to the foot. The authors speculate that it was the presence of two individuals that led to the female’s death, as she couldn’t escape, and it was the male who delivered the fatal injuries. They also conclude that the attacking female could not have successfully carried out this attack alone.

Although we cannot rule out the possibility that Walimah was attacked by another adult female orangutan, or that a female joined a male in an attack, we argue that this scenario is less likely than a solo attack by a male. First, female-female antagonism, and particularly direct contact, is rare in orangutans. In our study of female-female competition^103^ we recorded only 97 incidents of female-female aggression in 7041 orangutans follows (1.3%) and this was all non-contact aggression. Second, in the Tuanan case, the injured female had repeatedly been in antagonistic interactions with the attacking female^88^. We observed no cases of female harassment of Walimah in over 15,000 hours of observation. Third, no other injuries were found on Walimah. If the aim of the attack was lethal aggression against Walimah, rather than Walimah getting in the way of an attack on her infant, we would have expected additional injuries. While it is possible that Walimah herself was the victim of the attack and was unable to care for her infant after the injury, this seems unlikely due to the lack of additional injuries, such as seen in the Tuanan case. Infanticide by females is more common in mammalian species with intense resource competition^104^. While orangutans do experience some forms of resource competition^103,105^, it seems unlikely that a female orangutan was capable of carrying out this attack on her own. This is also apparently the case in chimpanzees where all cases of female infanticide have been carried out by a pair or group of females^106,107^. Fourth, as Marzec *et al*.^88^ found, instances of females attacking other females are very rare and are not known to cause severe injury, and they argue that a female would not have been able to deliver bites of the same force as a male. Indeed, the force of the bite needed to sever a portion of Walimah’s foot would have been substantial, but based on other orangutan injuries observed, is well within the capabilities of a flanged or unflanged adult male. Orangutans exhibit extreme sexual dimorphism, with flanged males being twice the size of adult females^108–110^. Unflanged males are closer to female size, but are still larger^111^. We also know from the regular occurrence of forced copulations that both types of male can easily overpower females^25^. Along with their larger bodies, males have substantially larger jaws and canines, with Bornean males having canines that are 1.48–1.63 times longer than those of females^112,113^. This evidence, combined with the semi-solitary nature of orangutans, makes it most likely that a single adult male orangutan carried out the attack.

We also argue that it is highly unlikely that Walimah was attacked by a male during a forced copulation. Orangutans have a mean inter-birth interval of 7.6 years^17^ and a mean interval of postpartum amenorrhea of 5.4 years^19^. This attack occurred 4 months after Walimah gave birth, many years before she would be expected to start ovulating again. Orangutan males typically avoid mating with females with very young infants, preferring to mate with reproductive females^98^. Furthermore, in an earlier review of forced copulations in wild orangutans, Knott^25^ found that although a male may attempt to restrain a female during forced copulations, which can include hitting and biting the female, there are no reports of bites ever breaking the skin. In fact, there are no reports of females receiving wounds of any kind, including soft tissue injuries.

However, injuries do sometimes occur in cases of sexually selected infanticide by males (Table 3), especially in great apes. Although in the majority of cases of primate infanticide mothers are not injured, this may be due to the fact that in some species males are more likely to attack infants who are not clinging to their mother’s body (e.g. Hanuman langurs, *Semnopithecus entellus*)^114^. However, Walimah’s infant was clinging 100% of the time before she disappeared, which is typical of ape infants less than 3 months of age^115–118^. In orangutan infants of 3–6 months of age, time spent off the mother’s body is minimal (21% of the time), always within the same tree and they are never more than 10 meters away^116^. Scott and Knott^119^ found that when males approach, the distance between mothers and infants also decreases, and no infant younger than 10 months was recorded out of contact with his or her mother when a male was present. Thus, an infanticide would have involved a very close association between the mother and the attacker, which would have increased the possibility of injury to the mother. Table 3 shows that when mothers are injured during successful infanticidal attacks, they are often injured on the extremities, consistent with the location of Walimah’s injury. Although rare, there are instances of male infanticide in primates where the male has taken an infant off of the female’s ventrum^7,120^. Based on the evidence, we conclude that the most probable cause of Walimah’s injury was a bite by a male orangutan during a successful infanticidal attack on her infant.

### Infanticide risk in primiparous females

Promiscuous mating by females serves to establish a nonzero chance of paternity among multiple males, thereby decreasing the risk of infanticide^2^. We suggest that in species where females mate with multiple males, primiparous mothers are at increased risk of infanticidal attack due to the fact that nulliparous females are not preferred as mating partners by males^121^, and thus they are less successful at employing promiscuous mating for paternity confusion as a counter-tactic to infanticide. Muller *et al*.^122^ found that male chimpanzees prefer mating with older, parous females over both nulliparous and young parous females, and suggest that this may be a result of accumulated maternal experience leading to lower rates of infant mortality for these mothers, or the possibility that their advanced age signals higher genetic quality. Anderson^121^ argues that the lack of male mating interest in primiparous females, across many primate species, is due to lower birth rates and lower infant survivorship for these inexperienced mothers. Additionally, primiparous females often have long periods of adolescent subfecundity^15,123–125^. The low probability of conception by these females may make them less attractive^122^ which may explain why males are not interested in mating with them. Orangutans, have a 4 year period of adolescent subfecundity^15^, likely the longest of any of the primates. As documented in other primates (chacma baboons, *Papio ursinus*^126^; chimpanzees, *Pan troglodytes*^127^; ring-tailed lemurs, *Lemur catta*^128^*;* mandrills, *Mandrillus sphinx*^129^), male orangutans seem to have low interest in mating with young nulliparous females^34,54,55^. Thus, if female orangutans rely on promiscuous mating as an anti-infanticide tactic^2,35^, nulliparous females, being less attractive to males, would have little opportunity to wield this tactic effectively. Without being able to effectively employ this behavioral counter-tactic, primiparous females should be at a higher risk of infanticidal attack compared to multiparous females.

Walimah’s case fits this prediction as she was a primiparous mother and was only seen mating with two males during her peri-conceptive period. As described, Walimah had long, elaborate, extremely proceptive sexual interactions with one flanged male, Codet, and comparatively short and notably less proceptive and even non-receptive matings with one unflanged male. Walimah displayed 7 months of high interest in Codet, before they successfully mated. We argue that because she invested so heavily in mating with this flanged male (Table 1), she did not mate with more of the other males in the study site. Given these observations of Walimah, along with our observations of other nulliparous female mating events that were similar, and the description Schürmann^54^ provided of nulliparous female and flanged male mating events, it seems that nulliparous female orangutans put forth a great deal of effort to mate with individual flanged males – effort that we do not see matched in parous females. Galdikas^34^ also found that nulliparous females were much more proceptive than parous females. Nulliparous female orangutans may be unable to sufficiently confuse paternity because they are unable to mate with as many males as parous females are, due to a combination of a lack of male interest in mating with them, their focus on flanged males, and their lack of social confidence^55^. We suggest that more studies, in primates and other species, should compare the ability of nulliparous versus parous females to mate promiscuously, and thus further test the hypothesis that primiparous mothers are at increased infanticide risk compared to multiparous mothers.

An interesting case of chimpanzee intragroup infanticide fits this prediction^130^. In this case, two females with 3–3.5 year old infants were present but only the primiparous female and her infant were attacked. Additionally, the primiparous female and infanticidal male were seen mating in the past, but at very low frequencies, when the female was maximally tumescent. This case supports the hypothesis that primiparous mothers may be at a disadvantage in terms of using promiscuous mating to protect their infants from infanticidal attacks by males.

### Infanticide in orangutans revisited

Although circumstantial, the evidence in this case points to an attack by a male orangutan, and is the first report of a suspected case of infanticide in wild orangutans. The circumstances also provide further evidence that although rare, infanticide may occur in orangutans. One of the arguments against the possibility of infanticide in orangutans has been that a male who killed a female’s offspring would be unlikely to have the opportunity to father subsequent offspring^46^. However, our data indicate that there is no reason to expect that a male would not be in the vicinity of a female several months after her infant’s death. Although we cannot say for certain who killed Walimah’s offspring, interestingly, she was seen with an unflanged male 23 days after she was found injured and without her infant. This male first appeared two weeks before she gave birth, was not present during Walimah’s peri-conceptive period and she had no known prior social interactions with him. During the month following the suspected infanticide, she was in almost continual consortship with this male. Matings were observed within 4 months of the suspected infanticide, although they may have occurred at an earlier date. Thus, the opportunity for a new male to enter the study area and then to kill an infant and subsequently sire the female’s next offspring existed. The assertion that a male who killed a female’s offspring would not have the opportunity to father future offspring with that female is not supported. Further, this timing of the return to mating after infant loss is comparable to the 2.5 month wait time reported by Galdikas^34^ after an ex-captive lost an infant, although ex-captive females may experience different energetic and social conditions. We have no way of knowing, if indeed Walimah and her baby were attacked by a male, if this was in fact *that* male. However, their subsequent consortship and mating demonstrate that a male of this species *could* have the opportunity to father a female’s offspring after infanticide, as predicted by the sexual selection hypothesis^2,29,32^.

Several other factors warrant the infanticide interpretation, and it fits the pattern observed in other great apes. Most intragroup infanticidal attacks by male chimpanzees occur during the first 6 months of an infants’ life^130,131^ and for gorillas, the mean age of infant victims was 358 days^132^. Walimah’s infant was approximately 4 months of age, consistent with when we might expect infanticide to occur. These first 6 months of lactation are also when we expect infant loss to have the greatest impact on female ovarian function, as it the most energetically demanding period for orangutan mothers^18^ and likely the most intense period of suckling. During this period Walimah may have been avoiding contact with conspecifics as she had no known social interactions during the period when she was with her new infant. Avoidance of potentially infanticidal conspecifics is another known anti-infanticide tactic employed by female mammals^30^. In a fission-fusion species such as orangutans, where individuals can choose whether or not to associate with others, this may be a preferred tactic. However, being alone also increases a female’s vulnerability, because of the lack of potential allies.

Our observations are thus consistent with the prediction that wild orangutan infants are vulnerable to infanticide^13,35^. It will be of some interest to see if further fieldwork produces additional data on this important topic in helping us to understand infanticide as a male reproductive tactic.

## Data Availability

The datasets generated during and/or analyzed during the current study are available from the corresponding author on reasonable request.
